# Impending sepsis-induced coagulopathy (SIC) is associated with increased disease severity in SIC-negative patients: a secondary analysis of a prospective exploratory study

**DOI:** 10.3389/fmed.2025.1525538

**Published:** 2025-02-17

**Authors:** Thomas Schmoch, Patrick Möhnle, Christian Nusshag, Manuel Feisst, Markus A. Weigand, Thorsten Brenner

**Affiliations:** ^1^Department of Anesthesiology and Intensive Care Medicine, University Hospital Essen, University Duisburg-Essen, Essen, Germany; ^2^Department of Anesthesiology and Intensive Care Medicine, Hôpitaux Robert Schuman – Hôpital Kirchberg, Luxembourg, Luxembourg; ^3^Department of Transfusion Medicine, Cell Therapeutics and Hemostaseology, Department of Anesthesiology, Klinikum der Ludwig-Maximilians-Universität, München, Germany; ^4^Department of Nephrology, Heidelberg University Hospital, Heidelberg, Germany; ^5^Institute of Medical Biometry, University of Heidelberg, Heidelberg, Germany; ^6^Department of Anesthesiology, Medical Faculty, Heidelberg University, Heidelberg, Germany

**Keywords:** sepsis, sepsis-induced coagulopathy, sepsis-associated coagulopathy, septic shock, scoring

## Abstract

Due to the intense crosstalk between the coagulation and immune systems, coagulation disorders are an integral part of the disturbed host response to infection that defines sepsis. These so-called sepsis-induced coagulopathies (SIC) are associated with increased morbidity and mortality. However, we still do not know enough about the prevalence and risk factors for SIC in different patient groups. In this study, we present a secondary analysis of a prospective, observational study. The objectives of this secondary analysis were (1) to estimate the prevalence of SIC at the onset of sepsis, (2) to assess the prevalence of SIC throughout the intensive care unit (ICU) stay using a previously described modified SIC score, and (3) to evaluate the association between SIC and morbidity and mortality. The prevalence of SIC at the onset of sepsis was 15.0% (95% confidence interval [CI]: 9.3–23.3%). A total of 24 additional patients who were SIC-negative at the onset of sepsis developed SIC according to the modified SIC score during their ICU stay. In total, we estimated that 39.0% (95% CI: 30.0–48.8%) of patients experienced relevant SIC during their ICU stay. Septic shock, a high lactate level, and a high Sequential Organ Failure Assessment (SOFA) score at the onset of sepsis in SIC-negative patients were associated with SIC development during the course of the disease. These findings need to be verified in larger cohorts and may represent a starting point for the development of a new screening tool for the identification of patients with sepsis at high risk of developing SIC.

## Introduction

Due to the intense crosstalk between the coagulation and immune systems, coagulation disorders are an integral part of the disturbed host response to infection that defines sepsis (1, 2). To objectify these coagulopathies and to be able to diagnose them as early as possible, two working groups coined two different terms in quick succession in 2017 and 2018 and provided diagnostic tools for their use (3, 4). One of these terms is “sepsis-induced coagulopathy” (SIC), introduced by the International Society on Thrombosis and Hemostasis (ISTH) (3, 4), and the other is “sepsis-associated coagulopathy” (SAC), introduced by Lyons et al. (3, 4) (Table 1) (3, 4). While the SIC score provides a dichotomous response (SIC negative or SIC positive), there are three degrees of severity of SAC: mild, moderate, and severe. Table 1 compares the SIC and SAC scores. The SIC score was introduced by the International Society on Thrombosis and Hemostasis (ITSH) in 2017 to provide a screening tool to detect coagulopathies that occur due to sepsis at an early stage, before overt disseminated intravascular coagulation (DIC) occurs (3). Almost at the same time, Lyons et al. (3, 4) introduced the SAC score to categorize the stages of coagulopathy that are associated with increasing mortality (Table 1). It should be noted that the criteria for mild SAC are more sensitive than those for SIC. Severe SAC, on the other hand, already describes an advanced, severe coagulation disorder. However, unlike SIC, severe SAC does not necessarily require a combination of an impaired INR and a low platelet count. A single severely disturbed value is sufficient for classification (Table 1).

**Table 1:** The SIC and SAC scores [modified from Schmoch et al. (5)].

SIC-score, Iba et al. (3)			SAC score, Lyons et al. (4)
		Points	
PLC SIC subscore (PSSC)	PLC ≥ 150/nL	0	Mild SAC 1.2 ≤ INR < 1.4 and 100 > PLC ≤ 150
	PLC 100 to <150/nL	1	
	PLC < 100/nL	2	
INR SIC subscore (ISSC)		0	
	INR 1.2 to < 1.4	1	
	INR ≥ 1.4	2	
SOFA subscore (truncated SOFA score including respiratory, cardiocirculatory, hepatic, and renal subscores)	1 point	1	
	2 points	2	Moderate SAC: 1.4 ≤ INR < 1.6 or 80 ≥ PLC ≤ 100;
No SIC		< 4	Severe SAC INR ≥ 1.6 or PLC < 80
SIC		≥ 4 (with PSSC plus ISSC >2/nL)	

INR, international normalized ratio; PLC, platelet count; SAC, sepsis-associated coagulopathy; SIC, sepsis-induced coagulopathy; SOFA, Sequential (Sepsis-Related) Organ Failure Assessment.

In 2023, we conducted a secondary analysis of two large randomized controlled trials and showed that the prevalence of SIC, which is significantly associated with mortality and morbidity, is 20–25% in patients with sepsis or septic shock (5–7). This was surprising because validation studies of the SIC scores from Japan (40–60%) and China (68%), as well as surveys of intensive care units (ICUs) in France (84.2%), reported significantly higher SIC prevalence’s (3, 8–12). The reason for this high variance remains largely unexplained. Indeed, a detailed comparison of the study cohorts from Japan, China, France, and Germany did not provide a sufficient explanation for the differences (5).

Here, we present a secondary analysis of the “Prediction of acute kidney injury with the need for renal replacement therapy by the use of cell cycle arrest biomarkers in patients with sepsis or septic shock” (PredARRT-Sep) trial (13). The objectives of this secondary analysis were (1) to estimate the prevalence of SIC and SAC at the onset of sepsis, (2) to assess the prevalence of SIC throughout the ICU stay using a previously described modified SIC score, and (3) to compare the association between three different subgroups (group I: no SIC throughout the ICU stay; group II: SIC at the onset of sepsis; and group III: SIC after the onset of sepsis) and the reported morbidity and mortality in another independent European cohort.

## Materials and methods

### Study population

In this study, we present a secondary analysis of a prospective, exploratory observational study conducted in two ICUs at the Heidelberg University Hospital (13). The study was conducted between May 2017 and July 2018 and included 100 patients with sepsis or septic shock (according to the Third International Consensus Definitions for Sepsis and Septic Shock [Sepsis-3]) and investigated whether the product of the two cell cycle arrest biomarkers—tissue inhibitor of metalloproteinase-2 and insulin-like growth factor-binding protein 7—and other innovative biomarkers could be used to predict sepsis-induced acute kidney injury requiring dialysis (2, 13). All patients were treated according to the guidelines of the Surviving Sepsis Campaign (SSC) that were valid at the respective time (14). Before the first patient was enrolled, the study was approved by the institutional review board and was registered in the German Clinical Trials Register (ID: DRKS00012446) (15). The trial was carried out according to the Declaration of Helsinki (October 2013), and written informed consent, including for secondary analyses, was obtained from all study participants (16).

### SIC score

The SIC score was calculated as suggested by Iba et al. (3) (Table 1). It was considered positive if two criteria were met simultaneously: (I) a total SIC score ≥ 4 and (II) the sum of the platelet count (PLC) SIC subscore (PSSC) and the international normalized ratio (INR) SIC subscore (ISSC) was ≥3 (3). The SIC score uses a truncated Sequential Organ Failure Assessment (SOFA) score that only takes into account the sum of the respiratory, cardiocirculatory, hepatic, and renal subscores (3, 17).

### Modified SIC-score

In our secondary analysis of the “Effect of Hydrocortisone on Development of Shock Among Patients With Severe Sepsis” HYPRESS trial, an increased PSSC reached a sensitivity of 84.8%, a specificity of 83.7%, a positive predictive value (PPV) of 59.5%, and a negative predictive value (NPV) of 91.1% for the prediction of SIC (5, 6). Therefore, we used a modified SIC score (SOFA score ≥ 2 and PSSC = 2) to estimate the prevalence of SIC after the onset of sepsis during the subsequent ICU stay as described previously (5).

### SAC score

The three severity levels of SAC were distinguished as described by Lyons et al. (4) (Table 1): mild SAC, 1.2 ≤ INR < 1.4 and 100 > PLC ≤ 150 [1/nL]; moderate SAC, 1.4 ≤ INR < 1.6 or 80 ≥ PLC ≤ 100 [1/nL]; and severe SAC, INR ≥ 1.6 or PLC < 80 [1/nL].

### Outcomes

The main outcome was SIC prevalence at the onset of sepsis. The secondary outcomes were SAC prevalence at the onset of sepsis and SIC prevalence during ICU stay. During the PredARRT-Sep trial, the lowest PLC sepsis onset (during the ICU stay) was documented. Using this number, we estimated SIC prevalence during the ICU stay using the described modified SIC-Score. Additionally, we divided patients into the following subgroups according to the development of their SIC status during the ICU stay: patients who were SIC negative at the onset of sepsis (SIC-Score) and stayed SIC negative (modified SIC-Score) during their entire ICU stay (group I: “no SIC throughout the ICU stay”), patients who were SIC positive already at the onset of sepsis (group II: “SIC at the onset of sepsis”), and patients who were SIC negative at the onset of sepsis (SIC-score) but became SIC positive (modified SIC-Score) during the ICU stay (group III: “SIC after the onset of sepsis”). We compared the following patient characteristics between the aforementioned subgroups: sex, age, source of infection, SOFA score at the onset of sepsis, serum lactate level, leukocyte count, C-reactive protein and procalcitonin levels, and the presence of septic shock at the onset of sepsis. Finally, we compared 28-day mortality, the need for mechanical ventilation during the ICU stay or renal replacement therapy up to day 7 after the onset of sepsis (as this was the recorded endpoint in the PredARRT-Sep trial), and ICU length of stay.

### Statistical analyses

We described the total cohort and the subgroups with appropriate measures of empirical distributions. Patient characteristics of subgroups were compared using the chi-square test, Fisher’s exact test, the Mann–Whitney *U*-test, the Kruskal–Wallis *H*-test, the Kaplan–Meier estimator, or the log-rank test, as appropriate. Point estimates of prevalence and rates, including diagnostic measures, are described by the relative frequency and the corresponding 95% confidence interval (CI) based on the Wilson/Braun method (18, 19). For all tests, two-sided calculations were carried out. All reported *p*-values have only descriptive meanings. We used GraphPad Prism 10 for Mac (Version 10.3.1; GraphPad Software, Boston, MA, United States) for statistical analysis and prepared the figures using Microsoft PowerPoint for Mac (version 16.89.1; Redmond, WA, United States).

## Results

### Prevalences of SIC and SAC

**Figure 1:**
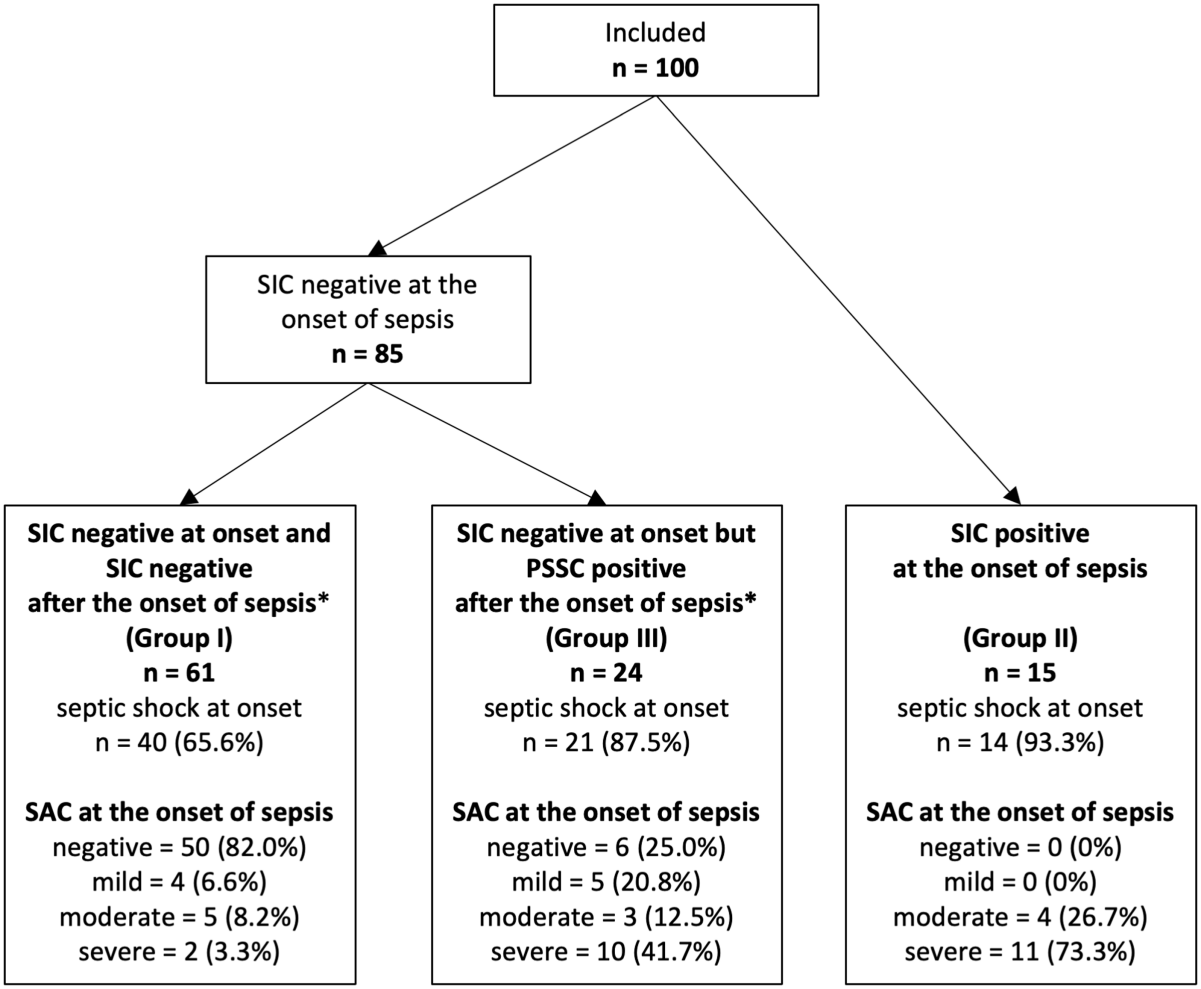
Flow diagram detailing the selection of patient groups analyzed from the PredARRT-Sep trial. PredARRT-Sep, Prediction of acute kidney injury with the need for renal replacement therapy by the use of cell cycle arrest biomarkers in patients with sepsis or septic shock; SAC, sepsis-associated coagulopathy; SIC, sepsis-induced coagulopathy.

All 100 patients who had been included in the PredARRT-Sep trial were eligible for our secondary analysis (Figure 1). The prevalence of SIC at the onset of sepsis (i.e., the day of sepsis diagnosis) was 15.0% (15 out of 100; 95% confidence interval [CI] 9.3–23.3%). During the ICU stay or within the 30-day observation period (whichever occurred first), 24 additional patients became SIC positive. Thus, 39.0% (95% CI: 30.0–48.8%) of patients experienced relevant SIC during their ICU stay. In contrast, the prevalence of SAC at the onset of sepsis was 44.0% (44 out of 100; 95% CI: 34.7–53.8%), specifically 9.0% for mild SAC (9 out of 100), 12.0% for moderate SAC (12 out of 100), and 23.0% for severe SAC (23 out of 100).

### Patient characteristics at the onset of sepsis

Group II patients, who were SIC positive at the onset of sepsis, had a higher median SOFA score, a higher serum lactate level, and were more often in septic shock at the onset of sepsis compared to group I patients, who were SIC negative at the onset of sepsis and remained SIC negative throughout their entire ICU stay (Table 2). When comparing group I patients with group III patients (who were SIC negative at the onset of sepsis but became SIC positive during their ICU stay), it became apparent that they already differed in terms of their disease severity at the onset of sepsis. Group III patients had a higher median SOFA score, a higher serum lactate level, and a higher procalcitonin level, and were more frequently in septic shock at the onset of sepsis (Table 2). Group II and group III patients were also more often SAC positive at the onset of sepsis. Thus, SAC positivity (mild, moderate, or severe) in SIC-negative patients at the onset of sepsis had a positive predictive value (PPV) of 62.1%, and SAC negativity had an NPV of 89.3% for identifying patients at risk of developing SIC during the ICU stay (Table 2).

**Table 2:** Sepsis onset characteristics for the three SIC subgroups.

Sepsis onset	Group I; SIC negative + PSSC < 2 after the onset of sepsis (*n* = 61)	Group II; SIC positive at the onset of sepsis (*n* = 15)	Group III; PSSC = 2 after the onset of sepsis (*n* = 24)	Total (*n* = 100)	*p*-value for group I vs. group II	*p*-value for group I vs. group III	*p*-value for comparison between all groups
Male subjects—no. (%)	32/61 (52.2%)	11/15 (73.3%)	18/24 (75.0%)	63/100 (63.0%)	0.14	0.06	0.11
Age [years]	66 [56–76]	67 [59–73]	66 [62–74]	66 [59–75]	0.67	0.48	0.75
Source of infection—no. (%)							
Pulmonary	22/61 (36.1%)	8/15 (53.3%)	13/24 (54.2%)	43/100 (43.0%)	0.22	0.13	0.21
Intraabdominal	39/61 (63.9%)	8/15 (53.3%)	16/24 (66.7%)	63/100 (63.0%)	0.22	0.81	0.45
Genitourinary	4/61 (6.6%)	2/15 (13.3%)	4/24 (16.7%)	10/100 (10.0%)	0.38	0.15	0.24
Other infection	7/61 (11.5%)	0/15 (0.0%)	0/24 (0.0%)	7/100 (7.0%)	0.17	0.08	0.17
SOFA [points]	11 [7–12]	16 [14–18]	14 [12–16]	12 [9–14]	**< 0.0001**	**< 0.0001**	**< 0.0001**
Serum lactate [mmol/L]	2.5 [1.9–4.8]	8.3 [6.1–13.9]	4.8 [3.4–10.0]	3.8 [2.2–6.6]	**< 0.0001**	**0.0004**	**< 0.0001**
Septic shock—no. (%)	40/61 (65.6%)	14/15 (93.3%)	21/24 (87.5%)	75/100 (75.0%)	**0.03**	**0.04**	0.03*
Inflammatory markers							
Leukocyte count [1/nL]	14.0 [9.3–20.0]	14.0 [8.3–21.0]	14.0 [8.3–21.0]	13.0 [7.9–22.1]	0.33	0.33	0.29
C-reactive protein [mg/L]	197.2 [147.9–312.5]	147.0 [98.9–215.9]	180.7 [142.5–264.5]	187.1 [144.5–301.7]	0.07	0.35	0.17
Procalcitonin [ng/mL]	4.9 [2.4–18.8]	22.8 [2.7–62.1]	19.7 [4.4–76.0]	7.1 [2.6–28.3]	0.11	**0.03**	**0.04**
SAC status at onset—no. (%)							
Negative	50/61 (82.0%)	0/15 (0%)	6/24 (25.0%)	56 (56.0%)	**< 0.0001**	**< 0.0001**	**< 0.0001**
Mild	4/61 (6.5%)	0/15 (0%)	5/24 (20.8%)	9 (9.0%)			
Moderate	5/61 (9.2%)	4/15 (26.7%)	3/24 (12.5%)	12 (12.0%)			
Severe	2/61 (3.3%)	11/15 (73.3%)	10/24 (41.7%)	23 (23.0%)			

Unless otherwise indicated, the data are presented as absolute frequencies and percentages in brackets, or as medians and [interquartile ranges]. PSSC, platelet sepsis-induced coagulopathy subscore; SAC, sepsis-associated coagulopathy; SIC, sepsis-induced coagulopathy; SOFA, Sequential (Sepsis-Related) Organ Failure Assessment. *p*-values < 0.05 are highlighted in bold.

### Clinical outcome parameters of different SIC subgroups

We found no statistically significant difference in 28-day mortality, the need for mechanical ventilation during the ICU stay, or ICU length of stay between group I and group II, or between group I and group III patients (Table 3). Of note, in comparison to group I patients, group II patients had a higher rate of acute kidney injury (AKI) requiring renal replacement therapy up to day 7 of their ICU stay (Table 3).

**Table 3:** Clinical outcome parameters.

	Group I; SIC negative + PSSC after the onset of sepsis < 2 (*n* = 61)	Group II; SIC positive at the onset of sepsis (*n* = 15)	Group III; PSSC = 2 after the onset of sepsis (*n* = 24)	Total (*n* = 100)	*p*-value for group I vs. group II	*p*-value for group I vs. group III	*p*-value for comparison between all groups
30-day mortality—no. (%)	14/61 (23.0%)	4/15 (26.7%)	10/24 (41.7%)	28/100 (28.0%)	0.69	0.09	0.25
Need for invasive mechanical ventilation—no. (%)	55/61 (90.2%)	14/15 (93.3%)	24/24 (100.0%)	93/100 (93.0%)	0.70	0.11	0.26
Need for renal replacement therapy up to day 7	6/57 (10.5%)	8/15 (53.3%)	6/22 (9.1%)	19/94 (20.2)	**0.0002**	0.06	**0.001**
Length of stay in intensive care unit [days]	19 [7–40]	23 [16–30]	15 [8–54]	20 [8–38]	0.65	0.84	0.95

Unless otherwise indicated, the data are presented as absolute frequencies and percentages in brackets, or as medians and [interquartile ranges]. SIC, sepsis-induced coagulopathy; SOFA, Sequential (sepsis-related) Organ Failure Assessment. *p*-values < 0.05 are highlighted in bold.

## Discussion

Within our presented secondary analysis of the PredARRT-Sep trial, the prevalence of SIC at the onset of sepsis (15.0%) appears to be comparable to the recently reported prevalence of SIC in the HYPRESS trial (16.9%) (5). However, we were able to show a higher prevalence of SIC (39.0%) throughout the sepsis-related ICU stay compared to both the HYPRESS (22.1%) and the “Effect of Sodium Selenite Administration and Procalcitonin-Guided Therapy on Mortality in Patients With Severe Sepsis or Septic Shock” (SISPCT; 24.2%) trials (5–7). This is not surprising given that SIC is a complication of sepsis, and it is especially associated with a highly severe form of the disease (2, 5, 12, 20). While the median SOFA score was 5 in the HYPRESS trial and 10 in the SISPCT trial (5), it was as high as 12 in the secondary analysis of our PredARRT-Sep trial. Of note, the prevalence of SIC was lower than the prevalence that had been reported from the SIC score validation studies (40–60%, based on a median SOFA score of <10 points) (3, 8–10). The only study that included patients with a comparably high sepsis severity (a median SOFA score of 11 points and patients with septic shock only) in ICUs in France recorded a prevalence of SIC of 84.2% (12).

Interestingly, the group of patients who were SIC-negative at the onset of sepsis but became SIC-positive during the course of the disease (group III) had the highest mortality rate by far. In particular, the difference in mortality between SIC-negative patients (group I) and SIC-positive patients after onset (group III) was clinically relevant, with a mortality difference of 19% (23% vs. 42%) at 30 days. The fact that this difference is not formally statistically significant is most likely an effect of the relatively high all-cause mortality in combination with the relatively small cohort size in the PredARRT-Sep trial. The average 30-day mortality in the PredARRT-Sep trial was 28%, while the 28-day mortality in the HYPRESS trial was only 8.5% (5, 6). Of note, early SIC (at the onset of sepsis) correlates with an increased need for renal replacement therapy until day 7 (post-onset), which, in turn, is consistent with the previously described association between SIC and increased morbidity (5).

The main strength of our study is that we analyzed a well-described cohort of 100 consecutively enrolled adult patients with a high sepsis disease severity. Except for the exclusion of patients with a preexisting need for renal replacement therapy, the PredARRT-Sep trial had no major exclusion criteria and, therefore, represents “real-world data” of an ICU with a high average severity of the disease. The major limitation of our study is the relatively small sample size, which makes it difficult to draw statistical conclusions from subgroup comparisons. Our results are only descriptive and hypothesis-generating and, therefore, need to be verified in larger cohorts.

Overall, our data from the HYPRESS, SISPCT, and PredARRT-Sep trials appear to indicate that we have to expect a somewhat lower prevalence of SIC in patient groups included in randomized interventional trials than initially suggested (3, 5, 8–10, 12). Interestingly, our secondary analysis of the PredARRT-Sep trial suggests a strong association between septic shock, a high lactate level, and a high SOFA score at the onset of sepsis with later SIC development in initially (at the onset of sepsis) SIC-negative patients. SAC positivity in SIC-negative patients at the onset of sepsis also appears to be strongly associated with later SIC development. However, it should be emphasized once again that this brief research report is an exploratory secondary analysis of a small cohort of 100 critically ill patients. Consequently, the observations presented here can only be of a hypothesis-generating nature. Using the data from the secondary analyses of the HYPRESS and the SISPCT trials, as well as the data from the PredARRT-Sep trial presented here, we have planned the “Incidence of Sepsis-Induced Coagulopathy” (INSIC) trial, an international, multicenter observational study to be conducted in March 2025 (5, 21). The plan is not only to measure the incidence and prevalence of SIC more accurately but also to collect information about its spontaneous course and to identify patients at high risk of developing SIC earlier and more effectively. Improved knowledge of the course of sepsis-induced coagulopathies may be useful as patients at risk could particularly benefit from specific treatment strategies—for example, an intensified anticoagulatory treatment. A retrospective study from Japan has already shown that patients with coagulopathy and patients with a high SOFA score (13–18 points) could particularly benefit from an intensified anticoagulatory treatment regimen (22). The findings of this brief research report could therefore contribute a small piece of the puzzle to the development of effective tools to predict the later development of SIC in patients who are SIC-negative at the onset of sepsis. However, these results can only serve as an initial hypothesis and a starting point for validation in larger studies.

## Data Availability

The data analyzed in this study is subject to the following licenses/restrictions: the datasets generated and/or analyzed during the current study are available from the corresponding author of the PredARRT-Sep-Trial, Christian Nusshag upon reasonable request. Requests to access these datasets should be directed to Christian Nusshag, christian.nusshag@med.uni-heidelberg.de.
